# Comparative Life Cycle Assessment of Asphalt Mixtures Using Composite Admixtures of Lignin and Glass Fibers

**DOI:** 10.3390/ma14216589

**Published:** 2021-11-02

**Authors:** Ahmed Khater, Dong Luo, Moustafa Abdelsalam, Jianxun Ma, Mohamed Ghazy

**Affiliations:** 1 School of Human Settlements and Civil Engineering, Xi’an Jiaotong University, Xi’an 710054, China; moustafa91@stu.xjtu.edu.cn (M.A.); majx@mail.xjtu.edu.cn (J.M.); 2 Department of Civil Engineering, Faculty of Engineering, Benha University, Benha 13512, Egypt; mohamed.ghazi@bhit.bu.edu.eg

**Keywords:** composite mixture, lignin fiber, glass fiber, life cycle assessment (LCA), environmental impacts

## Abstract

Lignin and glass fiber were used as additives to improve the quality of road pavements and minimize moisture damage and cracking at low temperatures on asphalt pavement, according to a previous laboratory study. The aim of this paper is to make a significant contribution to the environmental assessment of the construction of road pavements using four types of asphalt mixtures based on the life cycle assessment (LCA) methodology according to the requirements of ISO 14040, considering the impact of raw material extraction, asphalt mixture manufacturing, transportation, and wearing surface construction. The results of the environmental assessment showed that all studied asphalt mixtures do not offer any improvement in all impact categories, and three modified asphalt mixtures have a slight negative effect in all impact categories. The composite mixture has the highest negative effect of the studied three modified asphalt mixtures in all categories except in the marine aquatic ecotoxicity potential category and freshwater aquatic ecotoxicity potential category, where the lignin modified asphalt mixture has the highest negative effect in these two categories but has the best environmental impacts on most of other impact categories. Furthermore, the negative effect caused by composite asphalt mixtures is minimal and thus can be used to improve the overall performance of asphalt pavement.

## 1. Introduction

Road building may have detrimental environmental impacts and effects on air, water, and soil emissions [1]. The road construction industry is constantly pursuing technological changes that improve paving material performance, advance building efficiency, conserve energy, and enhance environmental protection [2]. The life cycle assessment (LCA) approach is one of the most commonly recognized and globally agreed approaches for evaluation the environmental effects of services/processes and determining their sustainability over the life cycle [3,4]. Both resource use and pollutant emissions related to the life cycle of a system or process are considered in the life cycle evaluation, such as the processing and extraction of raw materials, chemicals and fibers production, recycling, operation, and transport [5,6]. The LCA is very well developed and standardized at present [7]. It also contains a process of impact assessment during which all possible environmental effects are collected and quantified. This was achieved by defining and quantifying the materials and energy consumed and the environmental waste emitted and analyzing the possible environmental effects. The findings will support and guide decision-makers with regard to road pavements strategies.

Moreover, several kinds of raw materials are used by the road industry, such as aggregates, sand, bitumen, filler, and often selected additives that use a high quantity of natural resources and energy for the extraction, manufacturing, processing, and transport of raw materials. This industry is responsible for intensive emissions and contamination of air, water, and soil in the surrounding areas [8]. In China, about 290 million tons of CO_2_ emissions were produced by the highway industry in 2004, and the estimated emissions are expected to hit 1.1 billion tons by 2030 [9]. In addition to the asphalt mixture manufacturing and wearing surface construction processes, these emissions also occur through the extraction, manufacture, and transport of raw materials. Recently, the environmental impacts of road construction practices worldwide have been addressed in several LCA reports, but there are still several concerns that need to be further studied [10].

Several previous studies have been published on the application of LCA in road construction [11,12,13,14]. In research done by Ma et al. [15] using the LCA process, the environmental effects and resource use of hot mix asphalt (HMA) and warm mix asphalt (WMA) pavements were studied. The results showed that the lifetime resource use of HMA and WMA pavements was nearly at the same amount, whereas the environmental effects of the greenhouse gas related HMA pavement and PM_2.5_ emissions were slightly higher than those of the WMA pavement, except for the scenario where the long-term behavior of HMA pavement is much better than that of the WMA pavement. Another effort by Santos et al. [16] compared the possible environmental influences of the usage of polymer modified bitumen asphalt surface mixtures to those of a control bitumen surface mixture. It is obvious that the case of the usage of Ethylene Vinyl-Ac polymer as an additive result in a degradation of the pavement structure’s life cycle environmental profile in comparison to the use of conventional (without any additive) binder. This outcome differs from that in which waste nitrile rubber is used as an agent of the bitumen modifier, as it was proved to boost the environmental efficiency of the road pavement section’s life cycle. In the study obtained by Sackey et al. [17], a nano-silica-modified asphalt mixture (NMAM) over the LCA according to material output emissions was assessed, and to recognize the effective contribution of nano-silica in bitumen mixtures, the findings were compared to a control asphalt mixture. It showed that the global warming potential of NMAM was 7.45 × 10^3^ kg CO_2_-equivalent per unit of function as opposed to 7.42 × 10^3^ kg CO_2_-equivalent per traditional asphalt mixture unit of function. As reported by L. Vega A. et al. [18], the possible environmental effects of the usage of recycled concrete aggregate (RCA) in the manufacture of HMA as a fractional exchange of natural coarse aggregates were evaluated. It is obvious that mixtures containing 30% and 15% of RCA may be regarded as eco-friendly choices to the traditional mixture (i.e., without RCA content), as those two contents enabled decreases in every effect category rating. In contrast, a lower environmental efficiency than that of the traditional mixture was denoted by the mixture containing 45% of RCA.

## 2. Objective and Methodology

The LCA approach is used to capture the environmental consequences as well as the environmental advantages of a product, process, or system by evaluating the entire life cycle, according to ISO 14040 (2006a) and ISO 14044 (2006b). There are four basic steps in the LCA process: (1) defining goals and scope, (2) compiling life-cycle inventory, (3) evaluating the impacts, and (4) interpreting and analyzing the results [19].

In research done by Khater et al. [20], a laboratory study was done to evaluate the performance of asphalt mixtures using composite admixtures of lignin and glass fiber. The results showed that the addition of 0.30% lignin fiber and 0.30% glass fiber greatly enhanced the overall performance of bituminous blends, and the composite admixture was more effective for improving the asphalt performance than either lignin or glass fiber separately. Moreover, the results of the Marshall Immersion, freeze-thaw splitting, and low temperature bending tests for the behavior of the four types of asphalt mixtures, namely, the control asphalt mixture (C), lignin fiber modified asphalt mixture (L), glass fiber modified asphalt mixture (G), and a composite of lignin fiber and glass fiber modified asphalt mixture (LG) under the effects of water and low temperature are presented in Table 1. The study proved that the asphalt mixture reinforced with composite admixture showed significant improvement in the performance of moisture susceptibility and low temperature stability over other mixtures.

This study aims to explore the environmental impact of the use of the composite mixture of lignin and glass fibers used in the construction of wearing surfaces. The LCA methodology is used to compare selected types of modified asphalt mixtures from an environmental point of view. This contribution will be useful for decision makers when preparing and managing sustainable road development.

MSR is the ratio of residual stability; MS_2_ is the Marshall stability after 48 h of water immersion; MS_1_ is the Marshall stability of the fresh mixture after 30 min water immersion.

TSR is the tensile strength ratio (%); RT_2_ is the splitting strength of frozen-thawed samples (MPa); RT_1_ is the splitting strength of fresh samples (MPa).

The environmental impacts of road construction resulting from the extraction and production of materials, binders, and additives, in addition the manufacture of asphalt mixtures, the transport of materials and asphalt mixtures, as well as the construction of wearing surface, were measured in this study. All data on the background processes were obtained from the current Ecoinvent database (V 3.6, 2019) according to Chinese conditions and laboratory tests. SimaPro 9.1.0 software (Amersfoort, Netherlands) was used to determine the impacts of the environment for each pavement process. The environmental impacts related to the impact categories of abiotic depletion potential (ADP), acidification potential (AP), eutrophication potential (EP), global warming potential (GWP), ozone layer depletion (OLD), human toxicity potential (HTP), freshwater aquatic ecotoxicity potential (FWETP), marine aquatic ecotoxicity potential (METP), terrestrial ecotoxicity potential (TETP), and photochemical oxidant formation potential (POFP) are evaluated based on the CML2001 impact assessment methodology. These findings will play an important role in assisting industry and government decision-makers as a fundamental instrument in the development of road construction management strategies and policies, as well as in estimating investments in new road construction facilities.

### 2.1. Goal and Scope Definitions

#### 2.1.1. Purpose of the Study

The main aim of this study is to assess and compare the environmental consequences (using the LCA approach) of modified asphalt mixtures with lignin (and/or) glass fibers that were selected for the construction of the wearing surface layer of flexible pavements. Additionally, a comparison was made with the environmental impacts of the control asphalt mixture to afford a good understanding of the contribution effect of the selected additives in asphalt mixtures to provide information for decision-making.

#### 2.1.2. Functional Unit (FU)

The functional unit is the central core of any LCA study as it is a comparable unit in life cycle inventory. It provides a reference for each input, output, and environmental impact by using the same functional unit for different asphalt mixtures to give a more exact comparison.

**Figure 1:**
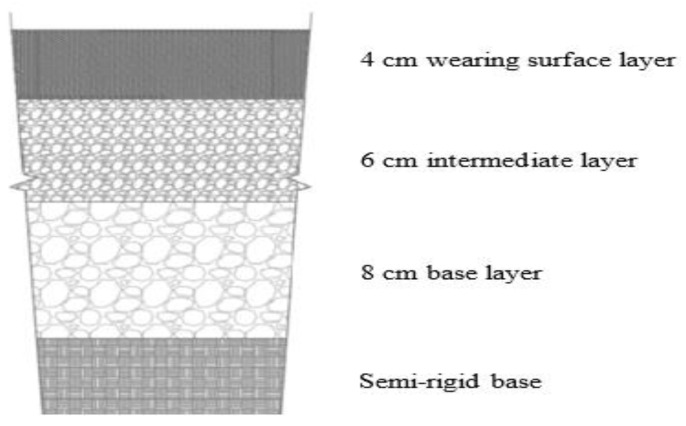
Section of asphalt pavement used in the study.

In this study, the functional unit is defined by the section of the wearing surface layer of typical pavement with a length of 1 km and 1 m width. The pavement was designed in terms of the conventional characteristics of traffic and subgrade support in China. The service life of the pavement, geometry, and performances present the main elements for the definition of function unit for road pavement LCA. The average annual daily traffic applied in this study is 20,000 vehicles/day with 8% heavy vehicles. The pavement is designed to serve for 15 years. The total thickness of the asphalt layer is 18 cm as shown in Figure 1; from top to bottom the pavement consists of three layers, which are 4 cm for wearing surface layer (AC-16), 6 cm asphalt concrete (AC-20), and 8 cm (AC-25), respectively. The mixtures were calculated from the Marshall design method according to the standard specification [21]. The boundaries for the pavement structure in this study were limited to the wearing surface layer. All emissions, materials, and energy consumptions are calculated according to this functional unit.

#### 2.1.3. System Description and Boundaries

It is very important to choose the system boundaries and parameters comprising the life cycle inventory as it mainly defines the goal of the study [22]. The system boundaries affect the final results of the study in addition to the interpretation of results [23].

**Figure 2:**
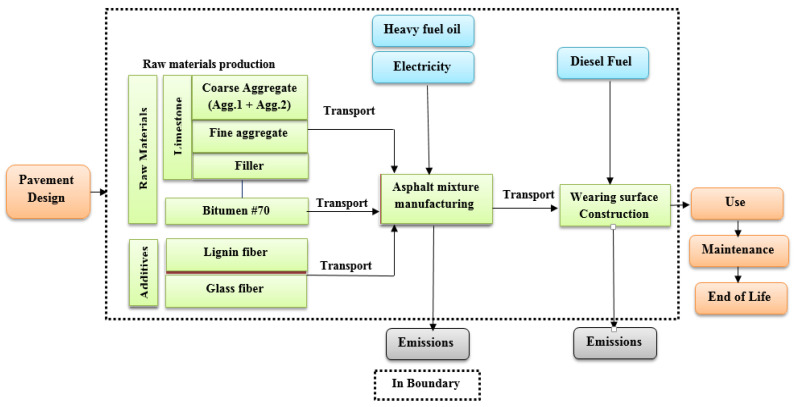
The system boundaries of selected pavement life cycle phases.

The life cycle of pavement construction consists of these processes: pavement design, raw material production, asphalt mixture manufacturing, transportation, pavement construction, use, maintenance, and end of life. The objective of the current study is mainly to compare the environmental impacts of the production and installation of the studied asphalt mixtures. The system boundaries of this study include only these four processes: raw material production, asphalt mixture manufacturing, transportation, and wearing surface construction. The system boundaries of selected pavement life cycle phases are presented in Figure 2 and described as follows:

- Raw materials production: The extraction of raw materials is considered the beginning step on the life cycle of the pavement. Essentially, the asphalt mixtures contain natural aggregates with different sizes, filler, asphalt binder (bitumen), and additives (lignin and glass fibers).
- Asphalt mixture manufacturing: All component materials of asphalt mixtures are first moved to the asphalt plant; after that, the aggregate is screened and dried, the asphalt is heated, and finally all components are mixed.
- Transportation: This phase includes the transportation of materials to the asphalt mixing plant after the process of raw material extraction. In addition, it includes the transportation of the mixtures to the pavement construction site after the process of asphalt mixtures manufacturing, as it is delivered to the construction site by the highway.
- Pavement construction: The construction of road pavement includes many processes, namely: clearing of the site, excavation, compaction of subgrade layer, and construction of the sub base layer, base layer, and wearing surface layer. The similar activities in the compared alternatives can be removed in LCA studies, so only the construction of the wearing surface layer process is considered in this study. The structure of the pavement shown in Figure 1 are compared among four asphalt mixtures with similar geometry but with different wearing surface layers. Each wearing surface layer for each asphalt mixture used different types of additive and different quantities of mixtures components.

#### 2.1.4. Data Sources and Quality

The classification and collection of data are considered the most important steps, as they consume more time and need the most effort in the LCA study. The data collected from the literature, database, pilot study, and experimental tests are the main sources for life cycle inventories.

In this study, the Ecoinvent database (V 3.6, 2019) established by the Swiss Centre for Life Cycle Inventory (LCI) was applied to evaluate the environmental impacts of most processes.

Mass balances for different asphalt mixture scenarios were calculated from experimental tests and using several data sources; they were modeled with a designed Excel spreadsheet in terms of the density and mix design evaluated from experimental tests for each asphalt mixture. Furthermore, the data on asphalt plant and paving machinery are collected from the Zhengzhou Sinosun Company in Zhengzhou, China and Shanghai DongMeng Road & Bridge Company in Shanghai, China.

#### 2.1.5. Assumptions and Limitations

This study has been conducted under the following limitations:

- The environmental impacts resulting from material loading onto the truck were neglected.
- The environmental impacts resulting from phases of pavement design, use, and end-of-life were not included in the system boundaries of this study, as the similar activities in the compared alternatives can be removed in LCA studies [24].
- The environmental impacts related to the transportation stage were considered for only the one-way trip of materials transportation to the asphalt mixing plant and asphalt mixtures transportation to the pavement construction site, whereas the return trips of empty trucks were ignored.
- The environmental impacts resulting from only the asphalt mixture manufacturing were considered, whereas the asphalt plant construction, including machinery and electric installation, were ignored.

## 3. Life Cycle Inventory (LCI)

The life cycle inventory defines the consumption of resources and energy and the environment emitted to water, land, and air during each pavement life cycle process. Treatment and measurement of data are essential steps in the LCA study for generating inventory data of various constituents or unit processes. Generally, data are also collected from different sources that may not be consistent with the current study functional unit and need to be edited to meet the study’s purpose. A comprehensive overview of the life cycle inventory of the input data used to model the different studied asphalt mixtures of the applicable system components is provided in this section in detail. For the asphalt mixtures production processes, the major design parameters and mass balances of ingredients are defined. In addition, all of the data on the background processes such as the transportation, supply of energy and material, resource extraction, and output of chemicals are taken from the existing Ecoinvent database (V 3.6, 2019) and listed briefly. To evaluate the impacts and burdens of the environment for each pavement phase, these data are employed in SimaPro 9.1.0 software that contains a detailed explanation of the following processes for the major inventory data. Furthermore, the APOS model was selected in this study. Since Ecoinvent Version 3 (2013) includes three system models (Cut-off) in addition to two new models called (APOS) and Consequential, where Version 2 had only a (Cut-off) system model. The Cut-off and APOS models’ outcomes are similar and depended on an attributional approach, and the mainly differ in terms of wastes and recycling materials. In contrast, the results of the Consequential model differ significantly from those of the attributional system models, which is to be expected because of fundamentally different modeling concepts that are based on the consequential method [25]. As the recycle process is excluded from all scenarios, the authors selected the APOS model for this Life Cycle Impact Assessment (LCIA) of this study.

### 3.1. Raw Materials Production Stage

The natural aggregates needed for the production of asphalt mixtures were modeled as limestone and the data of LCI relating to their extraction were obtained from the “Limestone, crushed, washed {RoW}| production | APOS, S” unit process of the Ecoinvent database. The LCI data associated with the bitumen production were also modeled from the Ecoinvent database in terms of “Bitumen, at refinery/kg/US”. The lignin fiber and glass fiber production information were taken from “Lignin fibre, inclusive blowing in {RoW}| production | APOS, S”, and “Glass fibre {RoW}| production | APOS, S” respectively, according to the Ecoinvent database.

### 3.2. Asphalt Mixtures Manufacturing Stage

This process aims to evaluate the environmental impacts resulting from the manufacturing of the different asphalt mixtures considered in the current study. The consumption of heavy fuel oil and electricity are essentially represented as the main causes for the consumption of energy during the manufacturing of the asphalt mixture. The electricity is consumed by the construction of the machinery that is fixed in the asphalt mixing plant, whereas the heavy fuel oil is consumed by the aggregate drying and heating of the bitumen. LCI data related to the production of electricity in China, obtained from the Ecoinvent database according to “Electricity, medium voltage, aluminium industry {CN}| electricity voltage transformation from high to medium voltage, aluminium industry | APOS, S”. The production of heavy fuel oil production data was taken from “Heavy fuel oil {RoW}| heavy fuel oil production, petroleum refinery operation | APOS, S” from the Ecoinvent database. Moreover, as listed in Table 2, the amount of electricity and heavy fuel oil needed for the production of 1 ton of asphalt mixture was collected from the Zhengzhou Sinosun Company in Zhengzhou, China.

### 3.3. Transportation Stage

In this process, the environmental burdens resulting from the transport of materials are generated by the pollutants released during the combustion process of transport vehicles that occurs during the trips from the extraction site of raw materials to the asphalt mixing plant and from the asphalt mixing plant to the construction site. The materials transportation process LCI data mainly depend on the type of vehicle and the distance traveled. Travel distances are essentially based on the circumstances of the local boundary. Ultimately, the high quantity of transported long-distance materials could have a direct effect on the energy balance of the system. Type of vehicle, categories of roads, distance to transport, and material weight are the main elements used to calculate the emissions and fuel consumption from the transport process. All raw materials and asphalt mixtures production were to be carried by heavy trucks, and the process used was “Transport, freight, lorry > 32 metric ton, EURO3 {RoW}| transport, freight, lorry > 32 metric ton, EURO3 | APOS, S” from the Ecoinvent database. Environmental impacts associated with the transport of raw materials and asphalt mixture production through the highway were assessed. Table 3 displays the applied distances for all the transport distances for raw materials and asphalt mixtures assumed in this study.

### 3.4. Wearing Surface Construction Stage

There are many processes in road construction, however, the wearing surface layer paving process is only included in this study. In addition, it was presumed that the paving process for all types of asphalt mixtures was similar. In this process, environmental impacts result from the emissions during the compaction and the spread of the asphalt pavement layers from the combustion of construction machinery. The data of LCI related to construction machines, such as a finisher and a heavy vibratory roller, which are only used in this process, were provided by Xuzhou Construction Machinery Group Company, and the energy consumed by such equipment is shown in Table 4. In the construction process of the wearing surface, the energy consumption is caused by the diesel fuel used by the construction machinery. The diesel fuel consumption related to the operation of these types of equipment was calculated by inserting the LCI data from the Ecoinvent database process “Diesel {RoW}|diesel production, petroleum refinery operation|APOS, S”.

### 3.5. Mass Balances for Different Asphalt Mixtures

The LCI for all processes and mass balance and energy of the four asphalt mixtures (control, lignin modified, glass modified, and composite) are calculated as shown in Table 5.

## 4. Life Cycle Impact Assessment (LCIA)

The LCIA process aims to recognize and calculate the extent and importance of any possible environmental impacts of a product or process during its life cycle.

### 4.1. Selection of LCIA Methodology and Impact Categories

In this analysis, the globally agreed problem-oriented approach is applied to remove the higher doubts of results depending on the methodological LCA guide provided by the Center of Environmental Science of Leiden University (CML2001 method). The main goal is to provide a relative comparison of the possible environmental consequences of the evaluated scenarios to estimate the alternative with the lowest environmental impacts. For the particular objectives and the area of this study, the CML2001 approach is more suitable because it can also be applied worldwide.

The selected impact categories in this study are listed in Table 6 and represent the baseline that was used in the LCIA process. These categories denote environmental impacts for pavement processes and depend on a well-recognized methodology [26].

### 4.2. Classification (Assignment of LCI Results)

In this process, the environmental interferences that are eligible and quantified in the analysis of inventory are allocated and combined into the aforementioned impact categories.

### 4.3. Characterization (Calculation of Category Indicator Results)

Characterization factors (science-based conversion factors) are applied in this phase to combine and convert the results of the LCI into an illustrative impact indicator for each selected impact category. The effects of the indicator category are determined by multiplying their consistent characterization factors by the related interferences of each category. Generally, a simple formula, as shown in Equation (1), can describe characterization processes [27].

(1) $${\mathrm{IR}}_{C}=\sum_{C}{\mathrm{CF}}_{\mathrm{CS}}*M_{S}$$

where IR_C_ is the impact indicator of category C; CF_CS_ is the characterization factor that attaches intervention S with impact category C; and M_S_ is the size of intervention S.

### 4.4. Normalization

In the characterization process, the normalized value of each impact category is calculated by dividing the outcome of each category indicated by the chosen reference value, as listed in Equation (2).

(2) $$N_{C}=\frac{{\mathrm{IR}}_{C}}{R_{C}}$$

where N_C_ is the normalized value of impact category indicator C; IR_C_ is the score of characterization indicator of category C; and R_C_ is the reference value of category C.

Based on [28], large normalization values compared to the total indicate the worst-performing categories; in contrast, those with small normalization values compared to the total indicate the better-performing categories. The normalized data used in the current study are based on the contribution of the world normalized data in 1995. These data are representative of the Chinese normalized data. The normalized data of the world in 1995 is presented in Table 7.

### 4.5. Weighting/Grouping

In this study, the Ecotax weighting method is conducted according to a mid-point monetary evaluation [30]. The Ecotax method of weighting factors was provided by Johansson [31]. This technique regularly depends on the resources and emission fees and taxes used in Sweden as a base of the economic values to provide mid-point evaluation-weighting factors. The Ecotax weighting factors resulting from environmental taxes and fees in Sweden 2002 are presented in Table 8.

## 5. Results and Discussion

### 5.1. Comparison of the Characterization Results

**Figure 3:**
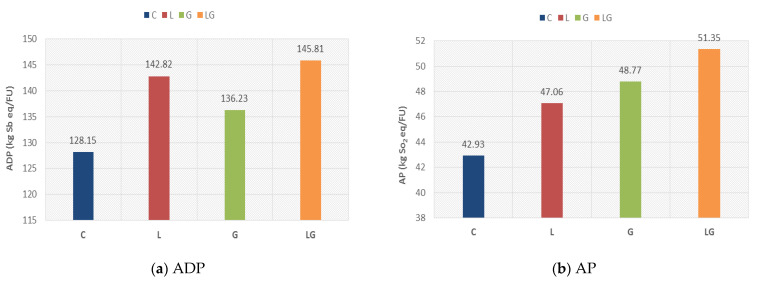

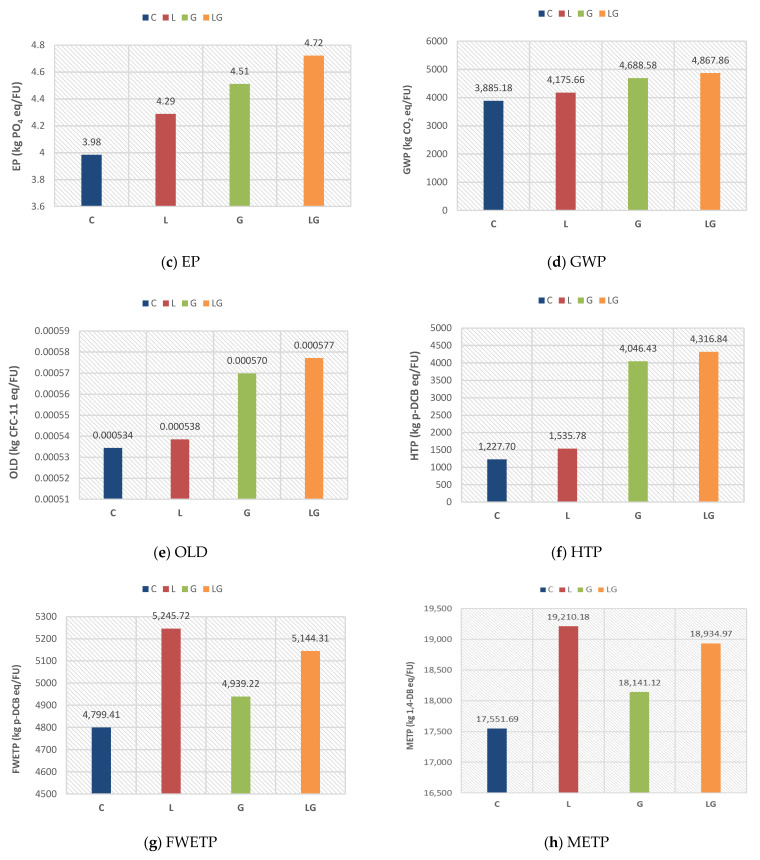

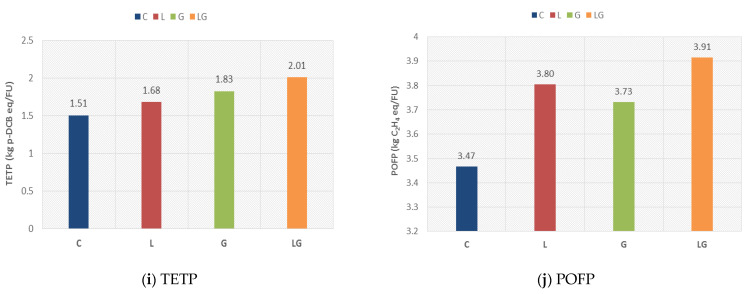
Characterization results of all impact categories.

The characterization results for all impact categories for different asphalt mixtures is presented in Figure 3 and Table 9. Results in Figure 3a demonstrate that the ADP value for asphalt mixtures modified by lignin fiber, glass fiber, and composite lignin and glass fiber increased by 11.4, 6.3, and 13.8%, respectively, when compared to the control, with the composite asphalt mixture exhibiting the worst results, as it has the highest ADP value.

In addition, Figure 3b reveals that the AP value of the mixture of lignin fiber, glass fiber, and composite mixture increased by 9.6, 13.6, and 19.6%, respectively, with respect to the control; furthermore, the composite asphalt mixture displays the worst results and has negative environmental impacts.

Figure 3c shows that the EP value increased with the addition of lignin fiber and glass fiber compared to control by 7.8 and 13.3%, respectively. Moreover, the composite additive increased the EP value by 18.6%.

Figure 3d demonstrates that the GWP value increased by 7.5, 20.7 and 25.3% for asphalt mixtures modified by lignin fiber, glass fiber, and composite lignin and glass fiber, respectively, when compared to the control, with the composite asphalt mixture exhibiting the worst results, as it has the highest GWP value.

Figure 3e reveals that the OLD value of the mixture of lignin fiber, glass fiber, and composite mixture increased by 0.75, 6.7, and 8.1% respectively with respect to control, and the composite asphalt mixture displays the worst results and has negative environmental impacts. The difference between the control mixture and the lignin modified mixture was very small.

It is observed in Figure 3f that the composite mixture has the highest HTP impact, and the control mixture has the lowest impact. The difference between the control mixture and the lignin modified mixture was very small, and the difference between these two mixtures and the other two mixtures was very large. Likewise, the HTP value increased with the addition of lignin fiber by 25.1% compared to control. Moreover, the composite additive and glass fiber increased the HTP value by 251.6 and 229.6%, respectively, compared to the control.

Figure 3g shows that the FWETP value increased by 9.3, 2.9, and 7.2% for asphalt mixtures modified by lignin fiber, glass fiber, and composite lignin and glass fiber, respectively, when compared to the control. The asphalt mixture modified with lignin fiber displayed the worst results, having the highest FWETP value.

Figure 3h demonstrates that the METP value increased with the addition of lignin fiber and glass fiber compared to control by 9.4 and 3.4%, respectively. Moreover, the composite additive increased the METP value by 7.9%.

Figure 3i reveals that the TETP value of the mixture of lignin fiber, glass fiber, and composite mixture increased by 11.3, 21.2, and 33.1%, respectively, with respect to the control. The composite asphalt mixture displayed the worst results, having negative environmental impacts.

It is observed from Figure 3j that the composite mixture has the highest POFP impact, whereas the control mixture has the lowest POFP impact. The POFP value increased with the addition of lignin fiber and glass fiber compared to control by 9.5, and 7.5%, respectively. Moreover, the composite additive increased the POFP value by 12.7% compared to the control.

The overall comparison of the characterization results for different asphalt mixtures showed that all studied asphalt mixtures do not offer any improvement in all impact categories. The three modified asphalt mixtures have a slight negative effect in all impact categories with a minimal difference from the control asphalt mixture. The exception is in the HTP impact categories for the glass modified asphalt mixture and composite mixture, which each have a large negative effect due to the presence of glass fiber. Furthermore, the composite mixture has the highest negative effect in all categories except in the METP and FWETP categories, where the lignin modified asphalt mixture has the highest negative effect. However, the results also showed that the different asphalt mixtures have the smallest negative impact on OLD. Likewise, all modified mixtures have a low negative effect in other impact categories.

**Figure 4:**
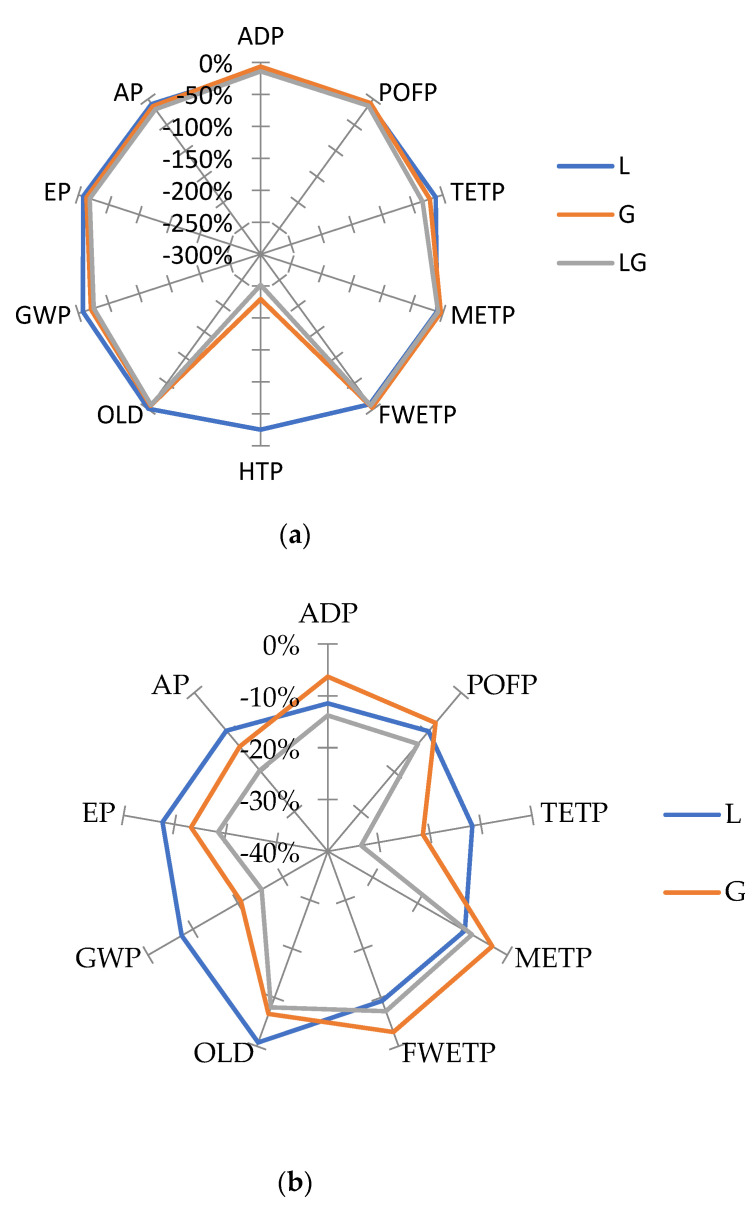
Overview comparisons of all asphalt mixtures: (**a**) for all impact categories; (**b**) for all impact categories except HTP.

Figure 4 presents the relative variation of the collective characterization results of different modified asphalt mixtures concerning those associated with the control asphalt mixture. The negative relative numbers represent worsening LCIA results on the studied asphalt mixtures compared to those associated with the control asphalt mixture.

The results demonstrate that the environmental impact of the HTP in the glass modified and composite mixtures was the lowest with −230% and −252%, respectively, due to the presence of glass fiber, followed by the TETP impact with −33% in the composite mixture. It is clear from Figure 4a that the impact category of the HTP dragged all values because of the extreme effect of glass fiber on it. Figure 4b shows that after excluding the results of the HTP category, the results of other impact categories became more obvious and the difference between their values is as shown. In summary, the composite mixture has the highest negative impacts for all impact categories except FWETP and MAETP. The lignin modified asphalt mixture has the highest negative impacts in these two categories, but it has the best environmental impacts in most of the impact categories except ADP and POFP, as the glass modified asphalt mixture has the best environmental impacts in these two categories.

### 5.2. Process Contribution Analysis

**Figure 5:**
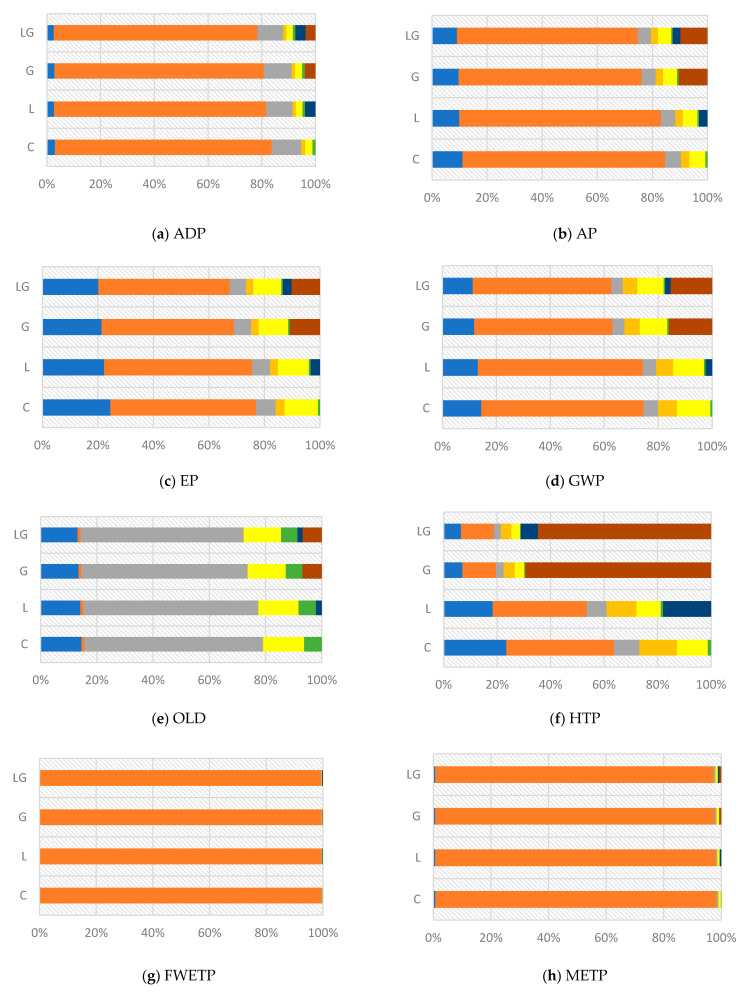

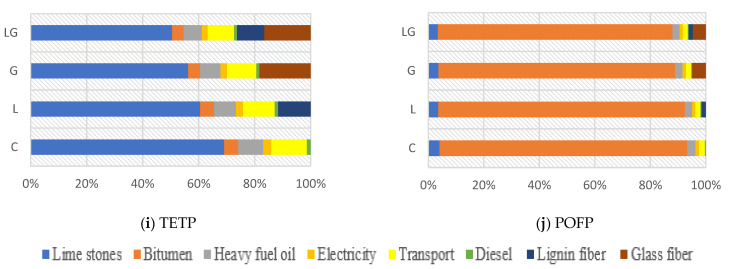
Relative contribution of the main processes to the total impact scores.

Figure 5 shows the contribution of the relative processes to the considered environmental impact categories of the different asphalt mixtures. The analysis of this figure revealed that the bitumen production, asphalt mixture manufacturing, glass fiber production, and aggregate extraction primarily drive the environmental impact profile of the different mixtures, whereas the variation of impact category is mainly responsible for the exact order. The bitumen production process has the highest contribution for the impact categories ADP, AP, EP, GWP, FWETP, METP, and POFP. Furthermore, the asphalt mixture manufacturing process has the highest contribution to the impact category OLD. In addition, the glass fiber production process has the highest contribution to the impact category HTP. Finally, the aggregate extraction process has the highest contribution to the impact category TETP.

### 5.3. Computation of Normalized Score

**Figure 6:**
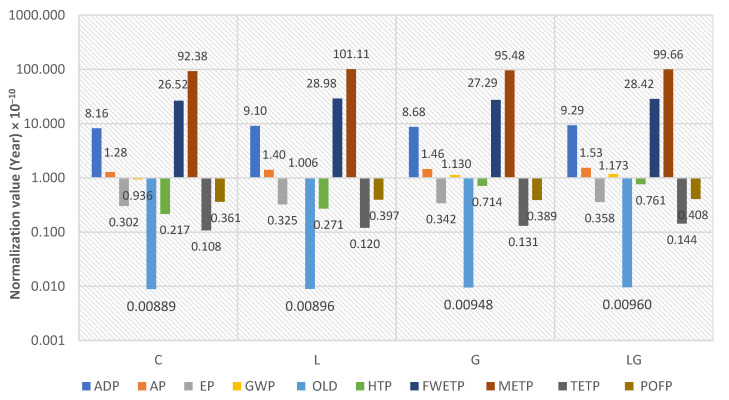
Normalization results of the different asphalt mixtures.

Figure 6 reveals that the METP category is the largest participant in all impact categories of the reference community, therefore it is considered the worst impact category compared to other categories, followed by FWETP and ADP. The results also proved that different asphalt mixtures only perform significantly better in four impact categories: EP, ODP, TETP, and POFP, as normalization of the characterization indicators showed a very limited share in these impact categories compared to the total environmental impacts of the reference community. In contrast, the AP and GWP categories had limited participation in the environmental impacts and the four asphalt mixtures had a slight difference in normalization among all impact categories.

An example for the normalized score calculation of the ADP impact category of the control mixture that was obtained from the characterization results indicated in Table 9, normalization factors world 1995 indicated in Table 7 and Equation (2) can be estimated as follows: (128.15/(1.57 × 10^11^)) = 8.16 Normalization value (Year) × 10^−10^.

### 5.4. Weighting and Grouping

**Figure 7:**
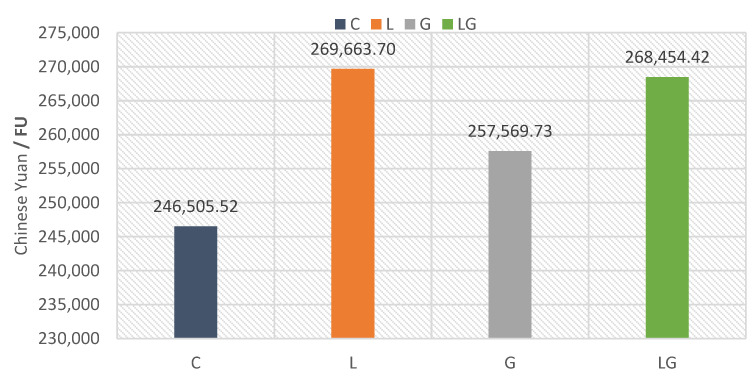
Weighting profiles of the different asphalt mixtures using the Ecotax2002 method.

The results of weighting according to the Ecotax 2002 method for the studied asphalt mixtures are shown in Figure 7. For example, the weighting score for the control mixture obtained from the characterization results in Table 9 and weighting factors of Ecotax 2002 method in Table 8 can be estimated as follows: (128.15 × 0.745) + (42.93 × 1.5) + (3.98 × 2.85) + (3885.18 × 0.063) + (0.000534 × 120) + (1227.70 × 0.15) + (4799.41 × 6.09) + (17,551.69 × 0.0606) + (1.51 × 17.6) + (3.47 × 48) = 31,085.18 Euro/FU= 246,505.5 Yuan/ FU.

The results showed that the weighting score increased by 9.39, 4.49, and 8.90% for asphalt mixtures modified by lignin fiber, glass fiber, and composite, respectively, when compared to the control. The asphalt mixture modified by lignin fiber has the highest environmental impacts of the compared mixtures. The composite mixture has the second-highest environmental impacts, followed by the asphalt mixture modified by glass fiber. The control was the best mixture that has the lowest environmental impacts of the comparing mixtures.

## 6. Conclusions

The environmental impact and contribution of additives in asphalt mixtures based on the LCA approach for the construction of the wearing surface layer of a Chinese road pavement section were analyzed, evaluated, and compared with a control asphalt mixture. The study examined three modified asphalt mixtures: lignin-modified, glass-modified, and composite. The life cycle of the road pavement manufacture process was split into four main stages: (1) raw materials production; (2) asphalt mixtures manufacturing; (3) transportation of materials; and (4) wearing surface construction. All of the data on background processes were taken from the existing Ecoinvent database (V3.6, 2019). To model and describe the environmental properties of the different asphalt mixtures, the SimaPro 9.1.0 program was used according to ISO 14040 guidelines. The major conclusions are summarized:

- All studied asphalt mixtures do not offer any improvement in all impact categories.
- The three modified asphalt mixtures have a slight negative effect in all impact categories with a minimal difference from the control asphalt mixture, except for the HTP impact category in glass modified asphalt mixture and composite mixture, followed by the TETP impact in the composite mixture.
- The composite mixture has the highest negative effect in all categories except in the METP and FWETP category, where the lignin modified asphalt mixture has the highest negative effect in these two categories, but the latter has the best environmental impacts on most of the other impact categories.
- The different asphalt mixtures have the smallest negative impact on OLD. Likewise, all modified mixtures have a low negative effect in other impact categories.
- The compared mixtures can be arranged as lignin modified mixture > glass modified mixture > composite mixture, based on their overall environmental impacts concerning those associated with the control asphalt mixture from the characterization results.
- The bitumen production process has the highest contribution for the impact categories ADP, AP, EP, GWP, FWETP, METP, and POFP. Moreover, the asphalt mixture manufacturing process has the highest contribution to the impact category OLD. In addition, the glass fiber production process has the highest contribution to the impact category HTP. Finally, the aggregate extraction process has the highest contribution to the impact category TETP.
- The studied asphalt mixtures can be arranged based on the weighting of their environmental impacts as a lignin modified mixture > composite mixture > glass modified mixture > control mixture.
- In summary, the negative effect caused by the composite asphalt mixtures and other modified mixtures is minimal related to their overall environmental impacts. Thus, the composite asphalt mixture can be used based on its overall enhanced performance advantages for the bituminous mixes.

## Figures and Tables

**Table 1:** Experimental results of different asphalt mixtures [20].

Asphalt Mixture Type	Marshall Immersion Test Results			Freeze-Thaw Splitting Test Results			Low Temperature Cracking Test Tesults	
	MS_1_ (kN)	MS_2_ (kN)	MSR (%)	R_T1_ (MPa)	R_T2_ (MPa)	TSR (%)	Bending Stress (MPa)	Bending Strain (µε)
C	10.89	9.29	85.3	0.684	0.544	79.51	8.20	2086.10
L	10.57	9.52	90.0	0.729	0.602	82.58	9.77	2601.66
G	10.16	9.67	95.1	0.747	0.649	86.82	9.70	2484.40
LG	10.80	10.67	98.8	0.765	0.675	88.22	10.37	3104.60

**Table 2:** Characterization of asphalt mixing plant.

Equipment Model	Installation Power (kW)	Rated Capacity (ton/h)	Capacity of Mixer (kg)	Fuel Consumption (kg/t)	Fuel Type
SAP100	232	100	1300	≤6.5	Heavy oil

**Table 3:** Transportation distances considered in this study.

Type of Material	From	To	One-Way Trip Distance (km)
Aggregates (Limestone)	Extraction and processing sites	Asphalt plant	30
Filler (Limestone)		Asphalt plant	30
Bitumen		Asphalt plant	80
Lignin fiber		Asphalt plant	100
Glass fiber		Asphalt plant	100
Asphalt mixture	Asphalt plant	Construction site	50

**Table 4:** The machinery and energy consumption of wearing surface construction stage.

Machine	Energy Consumption (L/1000 m^2^)	Fuel Type
Heavy vibratory roller	20	Diesel
Finisher	40	Diesel

**Table 5:** LCI for all processes and mass balance for the studied asphalt mixtures.

Process	Item	Amount				Unit	Database Process
		C	L	G	LG		
Raw material production	Coarse aggregate 1	24.52	24.00	24.23	23.94	ton	Limestone, crushed, washed {RoW}|production|APOS, S
	Coarse aggregate 2	33.35	32.64	32.95	32.56	ton	
	Fine aggregate	35.31	34.56	34.89	34.47	ton	
	Filler	4.90	4.80	4.85	4.79	ton	
	Bitumen	4.35	4.75	4.47	4.65	ton	Bitumen, at refinery/kg/US
	Lignin fiber	-	0.303	-	0.303	ton	Lignin fibre, inclusive blowing in {RoW}|production|APOS, S
	Glass fiber	-	-	0.305	0.303	ton	Glass fibre {RoW}| production | APOS, S
Asphalt mixture manufacturing	Heavy fuel oil	665.86	656.83	660.92	656.57	kg	Heavy fuel oil {RoW}|heavy fuel oil production, petroleum refinery operation|APOS, S
Asphalt mixture manufacturing	Electricity	237.66	234.44	235.90	234.34	kWh	Electricity, medium voltage, aluminium industry {CN}|electricity voltage transformation from high to medium voltage, aluminium industry|APOS, S
Wearing surface construction	Diesel fuel	59.5	59.5	59.5	59.5	kg	Diesel {RoW}|diesel production, petroleum refinery operation|APOS, S

**Table 6:** Impact categories and method of assessment.

Impact category	Units	LCIA Method
Abiotic depletion (ADP)	kg Sb eq	CML 2001
Acidification (AP)	kg SO_2_ eq	CML 2001
Eutrophication (EP)	kg PO_4_ eq	CML 2001
Global warming (GWP)	kg CO_2_ eq	CML 2001
Ozone depletion (OLD)	kg CFC-11 eq	CML 2001
Human toxicity (HTP)	kg p-DCB	CML 2001
Freshwater aquatic ecotoxicity (FWETP)	kg p-DCB	CML 2001
Marine aquatic ecotoxicity (METP)	kg p-DCB	CML 2001
Terrestrial ecotoxicity (TETP)	kg p-DCB	CML 2001
Photochemical oxidation (POFP)	kg C_2_H_4_ eq	CML 2001

**Table 7:** Normalization factor of the world in 1995 [29].

Impact Category	Reference Unit	Normalization Factor (R_C_)
Abiotic depletion (ADP)	kg Sb eq/year	1.57 × 10^11^
Acidification (AP)	kg SO_2_ eq/year	3.35 × 10^11^
Eutrophication (EP)	kg PO_4_ eq/year	1.32 × 10^11^
Global warming (GWP)	kg CO_2_ eq/year	4.15 × 10^13^
Ozone depletion (OLD)	kg CFC-11 eq/year	6.01 × 10^8^
Human toxicity (HTP)	kg p-DCB/year	5.67 × 10^13^
Freshwater aquatic ecotoxicity (FWETP)	kg p-DCB/year	1.81 × 10^12^
Marine aquatic ecotoxicity (METP)	kg p-DCB/year	1.9 × 10^12^
Terrestrial ecotoxicity (TETP)	kg p-DCB/year	1.4 × 10^11^
Photochemical oxidation (POFP)	kg C_2_H_4_ eq/year	9.59 × 10^10^

**Table 8:** Weighting factors of Ecotax 2002 method [32].

Impact Category	Reference Unit	Weight of Reference
Abiotic depletion (ADP)	kg Sb eq	0.745 Euro/kg
Acidification (AP)	kg SO_2_ eq	1.5 Euro/kg
Eutrophication (EP)	kg PO_4_ eq	2.85 Euro/kg
Global warming (GWP)	kg CO_2_ eq	0.063 Euro/kg
Ozone depletion (OLD)	kg CFC-11 eq	120 Euro/kg
Human toxicity (HTP)	kg p-DCB	0.15 Euro/kg
Freshwater aquatic ecotoxicity (FWETP)	kg p-DCB	6.09 Euro/kg
Marine aquatic ecotoxicity (METP)	kg p-DCB	0.0606 Euro/kg
Terrestrial ecotoxicity (TETP)	kg p-DCB	17.6 Euro/kg
Photochemical oxidation (POFP)	kg C_2_H_4_ eq	48 Euro/kg

The exchange rate is 1 Euro = 7.93 Chinese Yuan.

**Table 9:** LCIA results of different asphalt mixtures per FU.

Impact Category	Reference Unit	Impact Result			
		C	L	G	LG
ADP	kg Sb eq/FU	128.15	142.82	136.23	145.81
AP	kg SO_2_ eq/FU	42.93	47.06	48.77	51.35
EP	kg PO_4_ eq/FU	3.98	4.29	4.51	4.72
GWP	kg CO_2_ eq/FU	3885.18	4175.66	4688.58	4867.86
OLD	kg CFC-11 eq/FU	0.000534	0.000538	0.00057	0.000577
HTP	kg p-DCB/FU	1227.70	1535.78	4046.43	4316.84
FWETP	kg p-DCB/FU	4799.41	5245.72	4939.22	5144.31
METP	kg p-DCB/FU	17,551.69	19,210.18	18,141.12	18,934.97
TETP	kg p-DCB/FU	1.51	1.68	1.83	2.01
POFP	kg C_2_H_4_ eq/FU	3.47	3.80	3.73	3.91

## Data Availability

Data available on request due to restrictions, e.g., privacy or ethical. The data presented in this study are available on request from the corresponding author.
